# Clinical manifestations and outcomes in tubulointerstitial nephritis and uveitis syndrome: a case report and a systematic review in China

**DOI:** 10.1007/s11255-023-03797-6

**Published:** 2023-09-27

**Authors:** Jiazhen Shi, Shaoyan Xu, Jianxiang Chen, Henglan Wu

**Affiliations:** 1 Jiaxing University Master Degree Cultivation Base, Zhejiang Chinese Medical University, Hangzhou, Zhejiang China; 2 Department of Nephrology, The First Hospital of Jiaxing, First Affiliated Hospital of Jiaxing University, Jiaxing, 314000 Zhejiang China

**Keywords:** Tubulointerstitial nephritis, Uveitis, TINU syndrome, Glucocorticoid, Systematic review

## Abstract

**Purpose:**

Tubulointerstitial nephritis and uveitis (TINU) syndrome is an uncommon disease. We present a confirmed case of TINU syndrome, and a systematic review of epidemiological characteristics, clinical manifestations, management, and outcomes in Chinese patients.

**Methods:**

A systematic search was carried out using defined terms and updated up to September 2022, in PubMed, Web of Science, Wanfang, CNKI, and VIP, to identify reported cases of TINU in China, according to PRISMA guidelines.

**Results:**

An 18-year-old boy presented with elevated serum creatinine and 24-h urine protein level of > 2 g. Inspection result revealed acute tubulointerstitial nephritis, and bilateral uveitis. The patient was diagnosed with TINU syndrome and received treatment with methylprednisolone sodium succinate, which resulted in a significant decrease in creatinine and urinary protein levels. Systematic review identified 35 publications that met the inclusion criteria. A total of 71 cases were included in this article, of which 70 were from publications and 1 was from our hospital. The median age at onset was 42 years and was significantly lower in males than females (*P* < 0.05). The symptoms of uveitis often occurred after kidney injury (54%) and most uveitis was anterior (55%) and bilateral (75%). Among the 51 patients who were followed up for more than 6 months, 24 had recurrent ocular symptoms or progression to chronic uveitis. Twenty patients experienced chronic or progressive kidney disease.

**Conclusion:**

TINU syndrome is prone to misdiagnosis because kidney damage may not occur simultaneously with uveitis. The incidence of kidney sequelae in children is lower than that in adults, and glucocorticoids are the preferred treatment.

**INPLASY registration number:**

INPLASY202350050.

**Supplementary Information:**

The online version contains supplementary material available at 10.1007/s11255-023-03797-6.

## Introduction

Tubulointerstitial nephritis (TINU) syndrome, triggered by external factors, including drugs and microbial pathogens, is an autoimmune disease involving multiple systems [1]. In the past 45 years, approximately 600 such cases have been reported worldwide. Approximately 2% of patients with uveitis are diagnosed with TINU syndrome [2]. The pathophysiology of TINU syndrome is currently unclear. In 1985, circulating immune complexes were first identified in patients with TINU syndrome [3]. Subsequently, researchers found that the concentration of C4 was decreased in patients [4–6]. Therefore, humoral and cellular immunity are involved in its occurrence and development [7].

The TINU syndrome is characterized by tubulointerstitial nephritis and uveitis. The main manifestation of kidney is acute renal injury, and some patients may develop permanent renal injuries [8]. Eye symptoms can manifest in different forms include redness, eye pain, blurred vision, etc. The most common being bilateral anterior uveitis and may develop to a chronic [9]. Approximately 80 cases of TINU have been reported in China. Most reports are of single cases, with few clinical studies with large samples, and this have biased understanding of the pathogenesis of TINU syndrome, diagnosis, and the clinical efficacy evaluation of treatment methods. We performed a systematic review of previous case reports and small-sample observational studies in China to further study the epidemiological characteristics, clinical manifestations, diagnosis and treatment methods, and clinical outcomes of TINU syndrome.

## Methods

This review was performed in accordance with Preferred Reporting Items for Systematic Reviews and Meta-Analyses statements (PRISMA). Although there were two previous systematic reviews on TINU syndrome, involving western populations. We focus this systematic review on evaluation of the epidemiological characteristics, clinical manifestations, management, and outcomes of TINU syndrome reported in China. Improve awareness of TINU syndrome and reduce misdiagnosis rates.

### Search strategy

This systematic literature review on TINU syndrome adhered to the PRISMA guidelines. Articles were located by searching the terms ‘tubulointerstitial nephritis and uveitis’ OR ‘tubulointerstitial nephritis and uveitis syndrome’ OR ‘TINU syndrome’ OR ‘tubulointerstitial nephritis with uveitis’ OR ‘Dobrin syndrome’, AND ‘China’, in the following databases from inception to September 2022: PubMed, Web of Science, Wanfang, CNKI, and VIP (Supplemental appendix A).

We included studies related to TINU syndrome that met the Mandeville diagnostic criteria. If the two reports described the same patient(s), we only considered the more detailed publication. If reports were duplicated, the most recent publication was retained. We excluded reviews and cases that lacked demographic data, or were not from China.

### Data extraction

Two authors independently reviewed the titles, abstracts, and full texts of all retrieved articles and assessed whether the studies met the inclusion criteria. If there was a disagreement, a third reviewer was consulted.

All patient data included in the literature were classified and counted using an Excel table. The following data were collected: age, sex, pathogeny (infection, drug), prodrome (fever, fatigue, gastrointestinal symptoms, weight loss, and nocturia increase), eye symptoms (redness, pain, blurred vision, photophobia), type of uveitis, occurrence of the sequence of uveitis and TIN (uveitis occurs before renal injury, and both occur in parallel: the time between uveitis and renal injury is within 3 weeks; uveitis occurs after renal injury), treatment, follow-up time, and prognosis. In addition, the following laboratory parameters were collected: creatinine, urea, erythrocyte sedimentation rate (ESR), urinalysis, antinuclear antibodies (ANAs), C3, and C4.

We aimed to provide an overview of TINU symptoms, regardless of the risk of bias in the included studies. Therefore, the methodological quality of the included studies was not formally evaluated.

### Statistics

SPS software (version 25.0; SPSS Inc., Chicago, IL) was used for the statistical analysis. The measurement data conforming to the normal distribution is expressed by mean ± standard deviation, and the comparison between groups was conducted using the student’s *t*-test. The non-normally distributed measurement data were expressed as median and quartile intervals, and the inter-group comparison was conducted using the rank sum test. The counting data were described by the rate. When the theoretical frequency was ≥ 5 and *n* > 40, the inter-group comparison used the Chi-squared (*χ*^2^) test; when the theoretical frequency was less than 5 or *n* < 40, Fisher's exact probability method was used. Statistical significance was set at *P* < 0.05.

### Patient and public involvement

Not required.

## Results

### Case reports

The case report was conducted in accordance to the World Medical Association Declaration of Helsinki and approved by the Ethics Committee of Jiaxing First Hospital (2022-LY-411). The patient had signed informed consent forms.

An 18-year-old male patient was admitted to our hospital with a 1-month anorexia, accompanied by nausea, vomiting, and weight loss. The patient had been administered ceftriaxone and omeprazole for 15 days at a local hospital, without significant improvement.

**Fig. 1:**
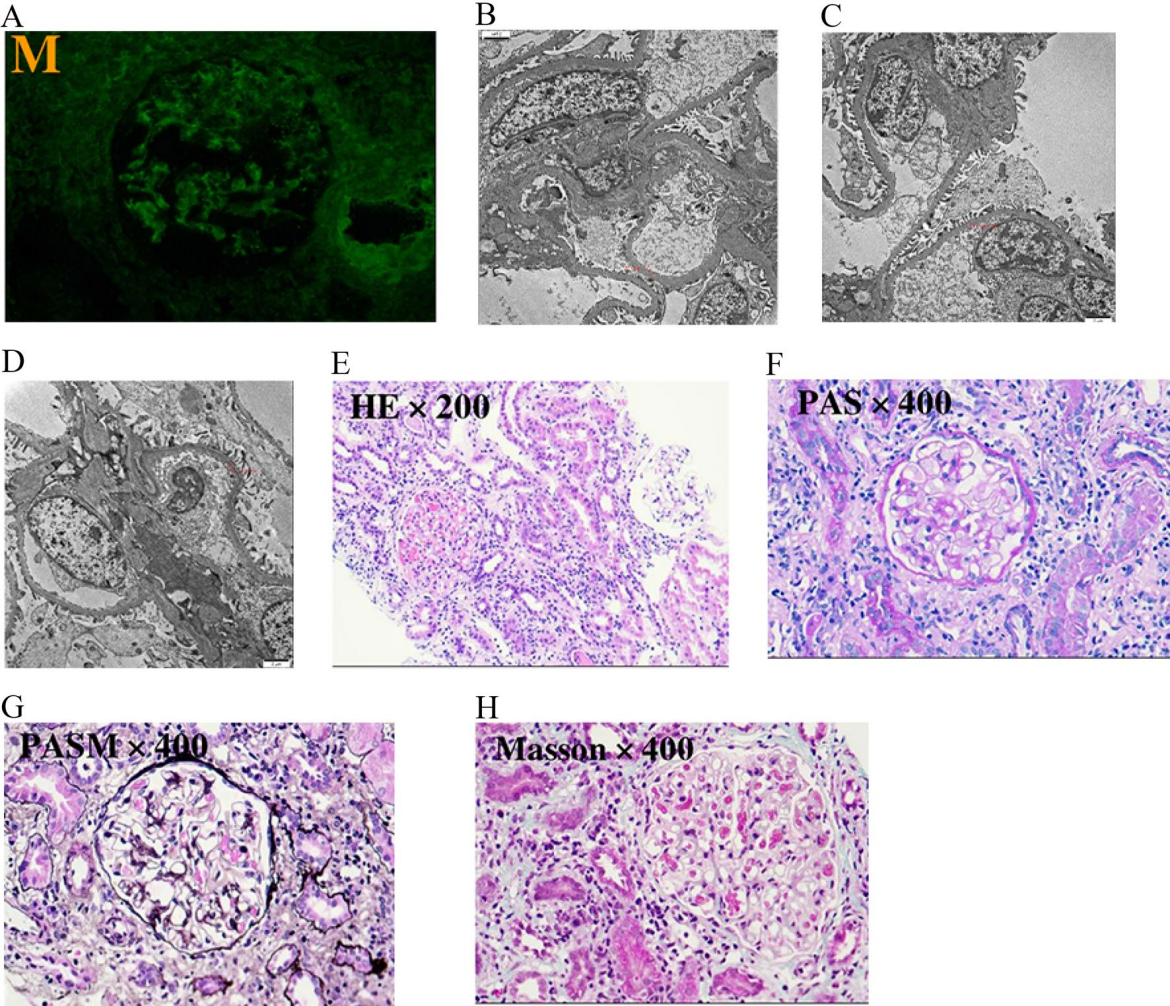
Results of renal biopsy. **A** Immunofluorescence: no immune complex deposit. **B**–**D** The electron microscope showed that the podocytes of the glomerulus were segmental fused, and there was no exact deposition of electronic dense matter in various parts of the glomerulus. (**E**) HE staining (×200). (**F**) PAS staining (×400). (**G**) PASM staining (×400). (**H**) Masson staining

Serum creatinine and urine protein levels increased within 3 months. Therefore, this patient was diagnosed with acute kidney disease. A detailed list of the paraclinical investigations is presented in Supplementary Appendix B. The kidney biopsy showed acute tubulointerstitial nephritis (Fig. 1).

**Fig. 2:**
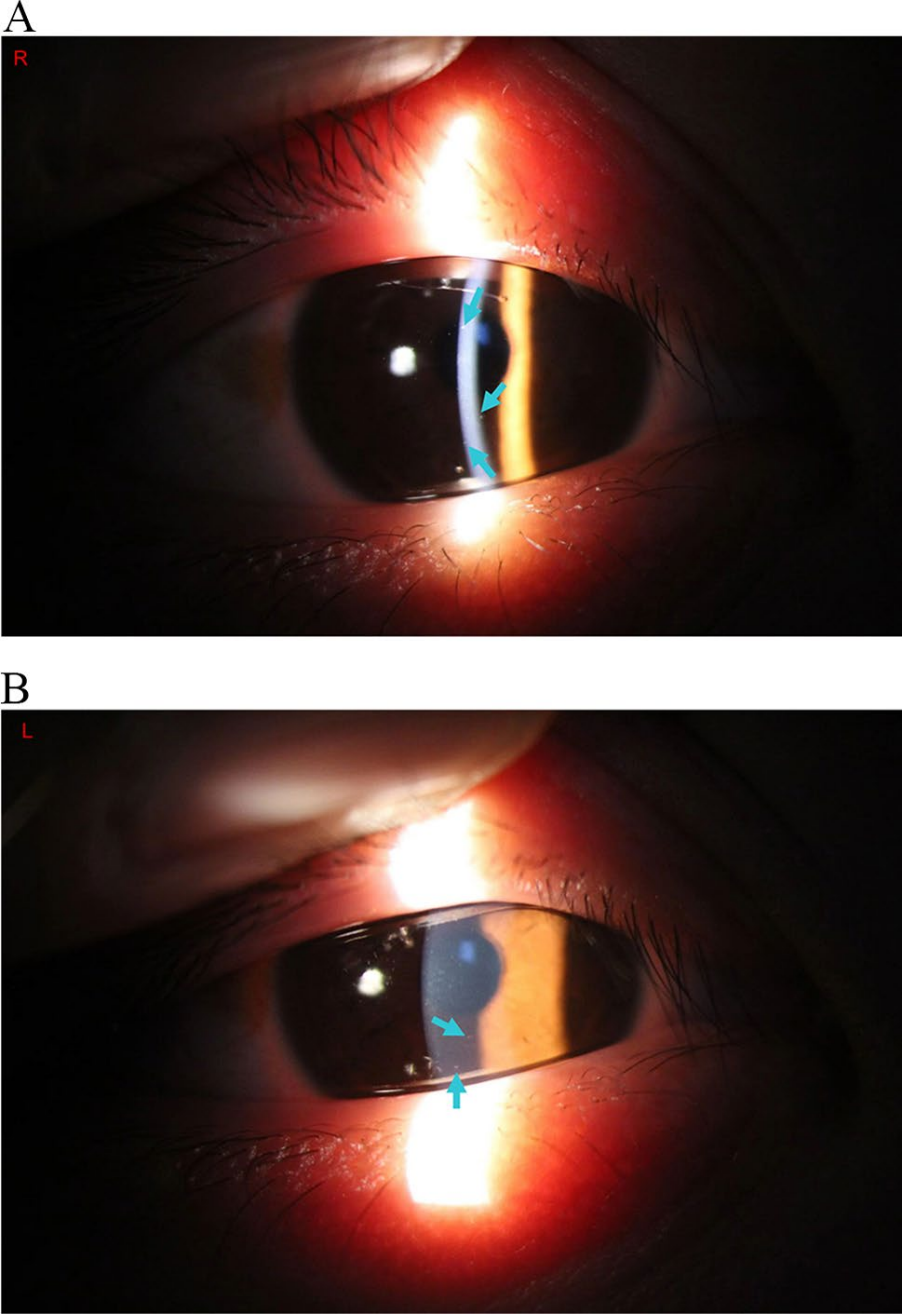
Slit lamp inspection results. (**A**) Right eye. (**B**) Left eye. The green arrow indicates keratic precipitates (KP)

The patient had no recent history of exposure to chemicals, drugs or foods that might cause acute renal injury. The patient reported redness and discomfort in his eyes during his preceding admission to a local hospital, which improved after 1 day. Accordingly, we consulted ophthalmologists and conducted slit-lamp examinations, which identified bilateral iridocyclitis (Fig. 2).

TINU syndrome was diagnosed, based on the renal pathology and the ocular lesions. Methylprednisolone (80 mg) was administered intravenously once a day for four consecutive days, and methylprednisolone tablets (48 mg) were administered once a day. All indicators improved after the treatment (Supplement Appendix B). During a follow-up period of > 5 months, the patient’s steroid therapy was discontinued, and there was no recurrence of renal damage or uveitis.

### Systematic review search results

**Fig. 3:**
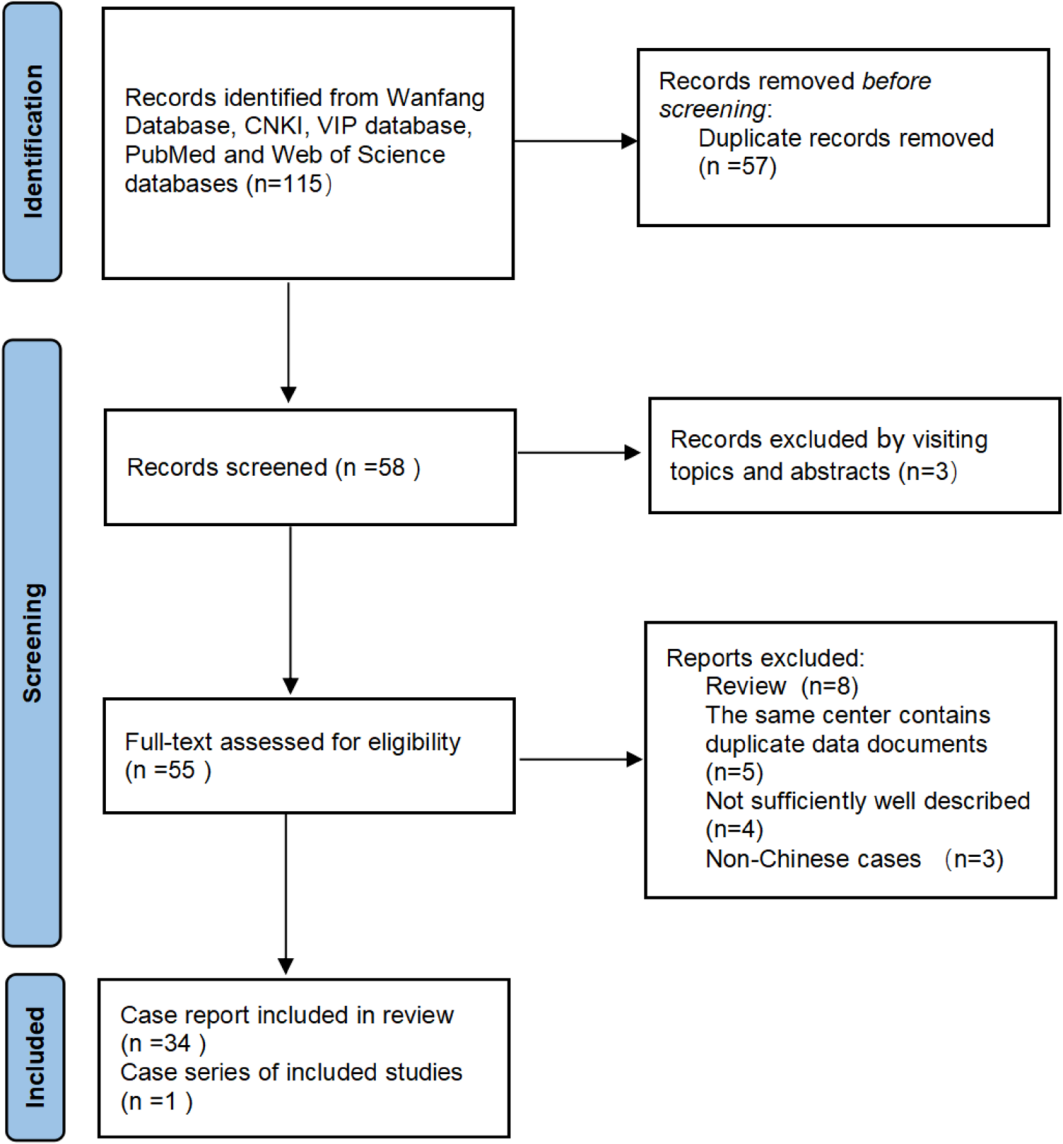
PRISMA study selection flowchart for systematic literature Review

A total of 115 relevant documents were retrieved; 57 duplicate documents were excluded, three documents were excluded because they are not related to the theme of this article after review of titles and abstracts, and 20 documents were excluded according to the exclusion criteria after visiting the full text (Fig. 3). Thirty-five publications met the inclusion criteria [10–44], comprising 34 case reports and one case series, totaling 70 cases. The characteristics of the included publications are summarized in Table 1.

**Table 1:** Characteristics of the included studies

Author	Age (years)	Gender	Eye symptoms	Urinalysis	Renal biopsy	Uveitis	Uveitis onset	Treatment	follow-up time(month)	Outcome
Wu Hua et al.	18	Male	Eye redness	Proteinuria Glycosuria Microscopic hematuria	Interstitial nephritis	Bilateral	After	Hormone Immunosuppressant	6	Recurrence of kidney disease or CKD
Liu Guangren et al.	14	Female	NA	NA	Acute interstitial nephritis	NA	Before	Hormone	36	Recurrence of kidney disease or CKD
Zhao Minghui et al.	74	Female	NA	Proteinuria Glycosuria	Acute interstitial nephritis	NA	After	Hormone	12	Recurrence of kidney disease or CKD Recurrence of uveitis or Chronic uveitis
Ma Yingchun et al.	28	Male	NA	Proteinuria Glycosuria Microscopic hematuria	Acute interstitial nephritis	Bilateral	After	Hormone	1	No recurrence
Zhang Minfang et al.	43	Female	Eye redness Blurred vision	Proteinuria	Acute interstitial nephritis	Bilateral	Before	Hormone	2	No recurrence
Li Yayu et al.	75	Female	Eye pain Blurred vision Photophobia	Glycosuria	NA	Bilateral	After	Hormone	0.5	No recurrence
Cheng Hongxin et al.	41	Female	Eye redness	Proteinuria Glycosuria	Interstitial nephritis	Unilateral	After	Hormone Immunosuppressant	NA	No recurrence
Zhang Wen et al.	56	Female	Eye redness Eye pain Photophobia	Proteinuria	Acute interstitial nephritis	Bilateral	Before	Hormone	1	No recurrence
Yao Yong et al.	10	Male	Eye redness Eye pain Photophobia	Proteinuria Glycosuria	Acute interstitial nephritis	Bilateral	After	Hormone	15	Recurrence of uveitis or Chronic uveitis
	15	Male	Eye pain Photophobia	Proteinuria Glycosuria	Acute interstitial nephritis	Bilateral	After	Hormone	19	Recurrence of uveitis or Chronic uveitis
Hu Junhua et al.	17	Male	Eye redness Blurred vision Photophobia	Proteinuria Glycosuria Microscopic hematuria	Interstitial nephritis	Unilateral	After	Hormone	2	No recurrence
	30	Female	Eye redness	Proteinuria Glycosuria Microscopic hematuria	Acute interstitial nephritis	Bilateral	Concurrent	Hormone	2	No recurrence
Chow,KM.et al.	30	Male	NA	Proteinuria	Acute interstitial nephritis	Bilateral	After	Hormone	NA	Recurrence of uveitis or Chronic uveitis
Liu Wenhui et al.	36	Male	Eye redness Eye pain	Proteinuria	Acute interstitial nephritis	Unilateral	After	Hormone	5	No recurrence
Zhou Letian et al.	15	Female	Eye redness Blurred vision	Proteinuria Glycosuria	Interstitial nephritis	Unilateral	Concurrent	Hormone	2	No recurrence
Tang Lin et al.	60	Female	Eye redness Eye pain Blurred vision	Proteinuria	Acute interstitial nephritis	Bilateral	Concurrent	Hormone	4	No recurrence
Hong Fuyuan et al.	52	Female	Eye redness Blurred vision	Proteinuria Glycosuria	Acute interstitial nephritis	Bilateral	Before	Hormone	2	No recurrence
Zhou, Letian et al.	15	Female	Eye redness Photophobia	Proteinuria Glycosuria	Acute interstitial nephritis	unilateral	Concurrent	Hormone	60	No recurrence
Yang Xiaochun et al.	52	Female	Eye redness Blurred vision	Proteinuria Glycosuria	Acute interstitial nephritis	Bilateral	Concurrent	Hormone Immunosuppressant	18	Recurrence of kidney disease or CKD
Ma Dong et al.	40	Female	Blurred vision	NA	NA	Unilateral	Concurrent	Hormone	0.5	No recurrence
Sun Dezhen et al.	41	Female	Eye redness Eye pain Photophobia	Proteinuria Glycosuria	NA	Bilateral	Before	Hormone	36	No recurrence
	37	Female	Eye redness Eye pain Photophobia	Proteinuria Glycosuria	Interstitial nephritis	Bilateral	Before	Hormone	36	No recurrence
	65	Female	Eye redness Eye pain Photophobia	Proteinuria Glycosuria	Interstitial nephritis	Unilateral	Concurrent	Hormone	24	No recurrence
Zhou Limei et al.	44	Male	Eye redness Blurred vision	Proteinuria	Interstitial nephritis	Bilateral	After	Hormone	0.5	No recurrence
Lei Wenhui et al.	60	Male	NA	Proteinuria Glycosuria Microscopic hematuria	Interstitial nephritis	Bilateral	Before	Hormone	6	No recurrence
Chen Min et al.	42	Female	Eye redness Eye pain Blurred vision Photophobia	Proteinuria Glycosuria	Interstitial nephritis	Bilateral	Concurrent	Hormone	0.5	No recurrence
Zhang Shu et al.	56	Female	Eye redness Photophobia	Proteinuria	Acute interstitial nephritis	Bilateral	Before	Hormone	7	No recurrence
Ren Wei et al.	16	Male	Eye pain Blurred vision	Proteinuria	Interstitial nephritis	Bilateral	Concurrent	Hormone	6	Recurrence of uveitis or Chronic uveitis
Zhou Yucong et al.	48	Male	Eye redness Eye pain Blurred vision	Proteinuria Glycosuria	Acute interstitial nephritis	Unilateral	Concurrent	Hormone	2	No recurrence
Lei Lei et al.	55	Female	Eye redness Blurred vision	Proteinuria	NA	Bilateral	After	Hormone	24	No recurrence
Wu Yao et al.	11	Male	Eye redness Eye pain Blurred vision	Glycosuria	Acute interstitial nephritis	Unilateral	After	Hormone	24	No recurrence
Guo Jingxiao et al.	11	Female	Eye pain	Proteinuria Glycosuria	Interstitial nephritis	Unilateral	After	Hormone	2	No recurrence
Kong Weiying et al.	37	Female	None	Proteinuria Glycosuria	Interstitial nephritis	Unilateral	After	Hormone	0.75	No recurrence
Wang Li et al.	63	Female	Eye redness	Proteinuria Glycosuria Microscopic hematuria	Interstitial nephritis	Bilateral	After renal involvement	Hormone Traditional Chinese Medicine	36	Recurrence of kidney disease or CKD Recurrence of uveitis or Chronic uveitis
Liu Jianliang et al.	38	Female	Eye redness Eye pain Blurred vision	Proteinuria Glycosuria	Interstitial nephritis	Unilateral	After	Hormone	1	No recurrence
Zhao	37	Male	NA	Proteinuria Glycosuria	Interstitial nephritis	Bilateral	NA	Hormone	24	Recurrence of kidney disease or CKD Recurrence of uveitis or Chronic uveitis
Xu Xialian et al.	64	Male	NA	None	Interstitial nephritis	NA	Before	Hormone	6	No recurrence
Zhang Kang et al.	49	Female	Eye redness Eye pain	Proteinuria Glycosuria	Acute interstitial nephritis	unilateral	Concurrent	Hormone Traditional Chinese Medicine	24	No recurrence
Yang Menglu et al.	Mean = 41	13 males and 19 females	28 Eye pain 28 Blurred vision and 16 Photophobia	NA	Interstitial nephritis	2 unilateral and 30 Bilateral	2 Before; 10 Concurrent and 20 After	13 Hormone therapy alone and 19 Hormone + Immunosuppressant	Mean = 37.8	18 Recurrence of uveitis or Chronic uveitis and 14 Recurrence of kidney disease or CKD

#### Demographic information

A total of 71 cases were included in this article, of which 70 were from publications and 1 was from our hospital. 38 were from Beijing, but there was no evidence of regional ethnic distribution differences in TINU syndrome in China. In 39 cases mentioning the triggering factors of TINU syndrome was: unclear (69%), infections (28%), drugs (3%). 11 patients had infectious triggers before acute kidney injury, of which nine patients had upper respiratory tract infection, one had cholecystitis, and one had urinary tract infection. Only one patients used herbal medicine which was not elaborated in the case report for 2 weeks before his serum creatinine found to be elevated. Twenty-eight patients were male and 43 were female, with a male-to-female ratio of 1:1.5 (Table 2). For those aged ≥ 18 years, 67% were female, while males accounted for 59% of those under 18 years of age. TINU syndrome tends to occur in young men and middle-aged women in China.

**Table 2:** Data of patients with TINU syndrome

Characteristics	*N*	Values
Gender (male:female), *n* (%)	71	28 (39):43 (61)
Age (years), median (IQR)	71	42 (18–55)
Pathogeny (s), *n* (%)	39	
None		27 (69)
Infection		11 (28)
Drugs		1 (3)
Prodromal symptoms, *n* (%)	71	
Fever		48 (68)
Fatigue		54 (76)
Gastrointestinal symptoms		40 (56)
Increased nocturia		11 (15)
Eye symptoms, *n* (%)	71	
Redness	33	26 (79)
Eye pain		44 (62)
Blurred vision		43 (61)
Photophobia		27 (38)
Uveitis, *n* (%)	71	
Anterior uveitis		39 (55)
Intermediate uveitis		9 (13)
Panuveitis		7 (10)
None		16 (23)
Uveitis (unilateral:bilateral), *n* (%)	71	15 (21):53 (75)
Uveitis onset, *n* (%)	70	
Before renal involvement		11 (16)
Concurrent renal involvement		21 (30)
After renal involvement		38 (54)
Urinary abnormalities, * n* (%)		
Proteinuria	37	34 (92)
Glycosuria	33	27 (82)
Microscopic hematuria	32	7 (22)
Treatment	71	
Glucocorticoid alone		47 (66)
Glucocorticoid + immunosuppressant		22 (31)
Other		2 (3)
The data of follow-up > 6 months	51	
Months, median (IQR)		24 (12–36)
Recurrence of uveitis or Chronic uveitis, * n* (%)		24 (47)
Recurrence of kidney disease or Chronic kidney disease, * n* (%)	42	20 (48)

#### Clinical and laboratory data

The patients had various symptoms, including fatigue, fever, and gastrointestinal manifestations such as nausea, vomiting, loss of appetite, and weight loss. Eleven patients urinate at least 2 times per night (Table 2). We conducted subgroup analysis according to sex and age. There were no statistically significant differences in clinical symptoms between men and women or in terms of age (Tables 3, 4).

**Table 3:** Comparison between male subgroup and female subgroup

Characteristics	Male	Female	*P* value
*n*	28	43	
Age (years), median (IQR)	29 (16–47)	50 (38–56)	< 0.05
Pathogeny (s), * n* (%)	15	24	
Unknown	11 (73)	16 (67)	0.734
Infection	3 (20)	8 (33)	0.477
Drugs	1 (7)	0 (0)	0.385
Prodromal symptoms, * n* (%)	15	24	
Fever	11 (73)	10 (42)	0.098
Fatigue	12 (80)	12 (50)	0.093
Gastrointestinal symptoms	7 (47)	11 (46)	1
Increased nocturia	2 (13)	7 (29)	0.437
Weight loss	3 (20)	3 (13)	0.658
Eye symptoms, * n* (%)	11	22	
Redness	8 (73)	18 (82)	0.661
Eye pain	6 (55)	10 (46)	0.721
Blurred vision	5 (46)	10 (46)	1
Photophobia	3 (27)	8 (36)	0.709
Uveitis, * n* (%)	15	24	
Anterior uveitis	6 (40)	14 (58.3)	0.333
Intermediate uveitis	1 (7)	1 (4.2)	1
Panuveitis	0 (0)	1 (4.2)	1
Unknown	8 (53)	8 (33.3)	0.318
Uveitis (unilateral:bilateral), * n* (%)	5 (18):22 (79)	10 (23):31 (72)	
Uveitis onset, * n* (%)	27	43	
Before renal involvement	3 (11)	8 (19)	0.616
Concurrent renal involvement	7 (26)	14 (33)	0.556
After renal involvement	17 (63)	21 (49)	0.248
Urinary abnormalities, * n* (%)			
Proteinuria	13 (87)	21 (96)	0.554
Glycosuria	10 (71)	17 (90)	0.363
Microscopic hematuria	5 (39)	2 (11)	0.091
Treatment	28	43	
Glucocorticoid alone	21 (75)	26 (61)	0.206
Glucocorticoid + immunosuppressant	7 (25)	15 (35)	0.379
The data of follow-up > 6 months	21	30	
Months, median (IQR)	24 (9–24)	30 (12–51)	
Recurrence of uveitis or chronic uveitis, * n* (%)	10 (48)	14 (47)	0.947
Recurrence of kidney disease or chronic kidney disease, * n* (%)	7 (39)	13 (54)	0.327

**Table 4:** Comparison between adolescent subgroup and adult subgroup

Characteristics	< 18 years	≥ 18 years	*P* value
*n*	17	54	
Gender (male:female), *n* (%)	10(59):7(41)	18(33):36(67)	
Pathogeny (s), * n* (%)	9	30	
Unknown	5 (56)	22 (73)	0.416
Infection	4 (44)	7 (23)	0.238
Drugs	0 (0)	1 (3.3)	1
Prodromal symptoms, * n* (%)	9	30	
Fever	5 (56)	16 (53)	1
Fatigue	8 (89)	16 (53)	0.115
Gastrointestinal symptoms	2 (22)	16 (53)	0.139
Increased nocturia	2 (22)	7 (23)	1
Weight loss	3 (33)	3 (10)	0.123
Eye symptoms, * n* (%)	8	25	
Redness	5 (63)	21 (84)	0.32
Eye pain	5 (63)	11 (44)	0.438
Blurred vision	4 (50)	11 (44)	1
Photophobia	4 (50)	7 (28)	0.391
Uveitis, * n* (%)	9	30	
Anterior uveitis	6 (67)	14 (47)	0.451
Intermediate uveitis	0 (0)	2 (7)	1
Panuveitis	0 (0)	1 (3)	1
Unknown	3 (33)	13 (43)	0.711
Uveitis (unilateral:bilateral), * n* (%)	5 (29):11 (65)	10 (19):42 (78)	
Uveitis onset, * n* (%)	17	53	
Before renal involvement	1 (6)	10 (19)	0.37
Concurrent renal involvement	3 (18)	18 (34)	0.201
After renal involvement	13 (77)	25 (47)	0.035
Urinary abnormalities, * n* (%)			
Proteinuria	7 (87)	27 (93)	0.53
Glycosuria	7 (88)	20 (80)	1
Microscopic hematuria	1 (13)	6 (25)	0.646
Treatment	17	54	
Glucocorticoid alone	17 (100)	30 (56)	< 0.01
Glucocorticoid + immunosuppressant	0 (0)	22 (41)	< 0.01
The data of follow-up > 6 months	14	37	
Months, median (IQR)	24 (14.25–36)	24 (12–48)	
Recurrence of uveitis or chronic uveitis, * n* (%)	6 (42)	18 (49)	0.712
Recurrence of kidney disease or chronic kidney disease, * n* (%)	2 (18)	18 (58)	0.023

Among the 35 patients with renal function data, all showed non-oliguric acute renal injury; average values of serum creatinine, urea nitrogen and 24-h urine protein were was 317.8 μmol/L, 13.7 mmol/L, and 1.14 ± 0.7 g/24 h, respectively. Increased ESR was reported in 26/27 patients. Proteinuria was present in 34/37 (92%) patients and Urinary glucose abnormalities with normal blood glucose levels developed in 27/33 (82%) patients. The renal biopsy results in 67 patients were consistent with acute tubulointerstitial nephritis (ATIN). Four patients who did not undergo renal biopsy had abnormal renal function, abnormal urine test results, and systemic disease lasting for 2 weeks.

#### Uveitis

In the 71 cases, anterior uveitis was found in 55%, intermediate uveitis in 13%, and panuveitis 10%. Bilateral uveitis was present in 53/71 cases. The patients exhibited various ocular symptoms, including redness, eye pain, blurred vision, and photophobia. The relationship between uveitis and ATIN remains unclear; 54% of the 70 patients had uveitis after ATIN, 30% had uveitis at the same time as ATIN, and 16% had uveitis before ATIN (Table 2). Subgroup analysis found that uveitis occurred after ATIN in 77% of those aged under 18 years, compared to 47% of adult patients (*P* < 0.05). There were not statistically significant differences in ocular symptoms between men and women (Tables 3, 4).

#### Treatments and outcomes

Glucocorticoid therapy was the first-line treatment for TINU in China. 47 patients were treated with glucocorticoids alone and 22 cases were treated with glucocorticoids combined with immunosuppressive agents, including mycophenolate mofetil (*n* = 10), cyclophosphamide (*n* = 8), azathioprine (*n* = 3) and unspecified (*n* = 1). All patients under 18 years received glucocorticoid therapy alone.

The outcomes of 51 patients with a follow-up period of > 6 months were analyzed. The median follow-up period was 24 months (IQR 12–36). Recurrent uveitis means that the eye symptoms of patients disappear after treatment, and then the eye symptoms reappear 3 months later. Recurrent uveitis or the course over 3 months defined chronic uveitis was present in 24/51 (47%) patients, comprising 10 males and 14 females, 6 aged less than 18 years and 18 adults. Forty-two out of 51 patients with a follow-up period of > 6 months for whom there was follow-up data on renal function, of which twenty patients experienced kidney disease recurrence or progression to CKD, including seven males and thirteen females. The incidence was higher in adults (58%) than in young adults (18%) (*P* < 0.05).

## Discussion

To the best of our knowledge, approximately 600 TINU cases have been reported worldwide. TINU syndrome has highly variable clinical features and a lack of awareness and recognition probably contributes to an underestimation of its incidence rate. The etiology and pathogenesis of TINU syndrome may be related to infection, chemicals, drugs (antibiotics and non-steroidal anti-inflammatory drugs), and genetic factors, such as human leukocyte antigen (HLA) [45]. There has been two systematic reviews about TINU syndrome before, involving western populations [46, 47]. Currently, there is no systematic review of TINU syndrome in China.

In the sample described here, the proportion of men and women with TINU syndrome in China was approximately 1:1.5, which is inconsistent with published articles [48, 49]. Nevertheless, this gender effect seems to be weakening in recent years. Regusci et al. reported that the male-to-female ratio was approximately 1:1.9, and in Japan, the proportion was approximately 1:2.3 [48, 50–52]. TINU syndrome is speculated to be a multisystem autoimmune disorder. The reason why women have an advantage may be related to their high levels of estrogen and immunoglobulin. Estrogen plays an important role in regulating innate immunity and is an enhancer of humoral immunity [53, 54]. And high levels of immunoglobulin will increase immune reactivity [55].

TINU syndrome has a variety of characteristic clinical manifestations, including fatigue, nausea and vomiting, fever, weight loss, and some patients have increased nocturia. Laboratory examination revealed non-oliguric acute renal injury with elevated serum creatinine and urea nitrogen levels. Urinalysis revealed mild-to-moderate proteinuria, urinary glucose abnormalities with normal blood glucose levels and renal tubular dysfunction. Renal injury and uveitis usually occur at different times, and uveitis can be diagnosed 2 months before or 12 months after interstitial nephritis (TIN) [51]. In our study, uveitis was diagnosed after TIN in 54% of cases, and most were anterior (55%) and bilateral (75%). This was very similar to the results of the systematic review in which reported that anterior accounts for 65% and bilateral accounts for 88% [46]. Consistently, Mandeville et al. reviewed the world's medical literature on TINU syndrome in 2001 and found bilateral at presentation in 77% of cases [51]. Uveitis in children was usually diagnosed after TIN (*P* < 0.05). These findings are consistent with data reported by Regusci et al. [46]. This is because children usually seek medical attention for nonspecific symptoms, and during this period, renal dysfunction is detected and diagnosed as TIN through renal biopsy. Subsequently, eye examinations were performed. In addition, the description of eye discomfort in children may be unclear and not been taken seriously by parents.

There are no standardized guidelines for the treatment of patients with TINU syndrome due to the lack of evidence; therefore, a prospective randomized controlled trial of glucocorticoids, placebos, and glucocorticoids combined with immunosuppressants is warranted. Glucocorticoid therapy is the first-line of treatment in China. Interestingly, subgroup analysis revealed that all children were treated with glucocorticoids alone, and the outcomes were good. It is recognized that the immune response of adolescents is not fully developed; therefore, the renal immune response damage caused by TINU syndrome is minimal in adolescents. This can also be used to explain the use of glucocorticoids alone can benefit children.

Previous studies have reported that TINU syndrome has a good renal outcome; however, our data show that approximately the half patient will progress to CKD [56, 57]. Among the forty-two patients with follow-up data on renal function, twenty individual experienced recurrence or progression to CKD. A study by Su et al. found that 63% of patients had estimated glomerular filtration rate (eGFR) < 60 mL/min/1.73 m^2^ after 3 years of follow-up [58]. The difference between the data in the previous literature and the newer results may be related to the length of follow-up. Our subgroup analysis showed that children underwent renal recovery more frequently than adults (*P* < 0.05). This is consistent with the results of previous studies [48–50]. A study suggest age at onset was associated with an increased risk of CKD development [46].

Our systematic review analyzed patients with TINU syndrome reported in the Chinese literature and we analyzed the renal and ocular outcomes of TINU syndrome, increasing people's understanding of TINU syndrome. Some of the included publications did not fully describe the clinical data, which may have led to bias in the results. The duration of follow-up varied, which may have affected the reported outcomes. This systematic review is a preliminary exploration of the TINU syndrome in China and needs to be further confirmed in a well-designed prospective study.

## Conclusion

We present a case of acute renal tubulointerstitial nephritis and bilateral uveitis, with a diagnosis of TINU syndrome. TINU syndrome affects predominantly adults in China. The etiology, clinical manifestations, and duration of uveitis and kidney injury are unclear, which increases the risk of missed diagnosis of the disease. When a patient's examination indicates acute renal injury with tubule dysfunction and excludes other diseases, it is necessary to consider the possibility of TINU syndrome. Simultaneously, doctors must carefully inquire about the eye symptoms and conduct an ophthalmic examination. Once confirmed, appropriate treatment strategies should be selected based on the patient's condition. Glucocorticoid therapy is currently the first-line treatment in China.

### Supplementary Information

Below is the link to the electronic supplementary material.

Supplementary file1 (DOC 40 KB)

## Data Availability

All data relevant to the study are included in the article or uploaded as supplementary information.
