# Supplementary material for: Clinical manifestations and outcomes in tubulointerstitial nephritis and uveitis syndrome: a case report and a systematic review in China
Source: Int Urol Nephrol. 2023 Sep 27;56(4):1415–27. doi: 10.1007/s11255-023-03797-6 (PMC10924013; doi:10.1007/s11255-023-03797-6)
Supplement: Supplementary file 1 — Supplementary file1 (DOC 40 KB) [file 11255_2023_3797_MOESM1_ESM.doc]

**Appendix A. Search Strategy**

Search date: 28 sep2022

**PubMed**

(“Tubulointerstitial nephritis and uveitis” [Mesh] OR “Tubulointerstitial nephritis AND uveitis syndrome” [tiab] OR “TINU syndrome” [tiab] OR “Tubulointerstitial Nephritis with Uveitis” [tiab] OR “Dobrin syndrome ” [tiab] ) AND (“china” [Mesh] OR “Mainland China” [Affiliation])

**Web of science**

TS=(“Tubulointerstitial nephritis AND uveitis” OR “Tubulointerstitial nephritis AND uveitis syndrome” OR “TINU syndrome” OR “Tubulointerstitial Nephritis with Uveitis” OR “Dobrin syndrome”) AND AD=(“China” OR “Mainland China”)

**Wanfang**

KW (‘Tubulointerstitial nephritis and uveitis’ OR ‘Tubulointerstitial nephritis AND uveitis syndrome’ OR ‘TINU syndrome’ OR ‘Tubulointerstitial Nephritis with Uveitis’ OR ‘Dobrin syndrome’)

**CNKI**

KW (‘Tubulointerstitial nephritis and uveitis’ OR ‘Tubulointerstitial nephritis AND uveitis syndrome’ OR ‘TINU syndrome’ OR ‘Tubulointerstitial Nephritis with Uveitis’ OR ‘Dobrin syndrome’)

**VIP**

TI, KW (‘Tubulointerstitial nephritis and uveitis’ OR ‘Tubulointerstitial nephritis AND uveitis syndrome’ OR ‘TINU syndrome’ OR ‘Tubulointerstitial Nephritis with Uveitis’ OR ‘Dobrin syndrome’)

**Appendix B. Laboratory examination results of a patient with TINU syndrome followed up over 5 months.**

|  |  | 7.6 | 7.25 | 8.29 | 9.12 | 11.12 | 2023.1.4 |
| --- | --- | --- | --- | --- | --- | --- | --- |
|  | Blood creatinine（umol/L） | 213.3 | 118.9 | 88.4 | 82.2 | 94.4 | 98.7 |
| urinalysis | Urine protein | 1+ | +- | - | - | - | +- |
|  | Urine glucose | 1+ | - | - | - | - | - |
| Five items of urine protein | transferrin（mg/L） | 8.79 | 5.61 | 2.35 | 1.49 |  | 2.72 |
|  | ImmunoglobuliG（mg/L） | 43.88 | 21.42 | 11.49 | 10.37 |  | 16.9 |
|  | α1 Microglobulin（mg/L） | 161.51 | 84.93 | 68.28 | 54.38 |  | 40.24 |
|  | Microalbumin（mg/L） | 113.4 | 40.6 | 19.7 | 16.6 |  | 30.2 |
|  | β2 Microglobulin（mg/L） | 32.33 |  |  | 2.43 |  |  |
|  | 24-hour urine protein（mg/24h） | 1001.3 | 270 | 213 |  |  |  |
